# Perception of Violence by Psychiatric Nurses: Behind the scenes

**DOI:** 10.1192/j.eurpsy.2024.1236

**Published:** 2024-08-27

**Authors:** S. Hamzaoui, K. Mahfoudh, S. Walha, D. Mezri, A. Ouertani, U. Ouali, A. Aissa, R. Jomli

**Affiliations:** Psychiatry A, Razi Hospital, Manouba, Tunisia

## Abstract

**Introduction:**

Violence in psychiatric settings poses significant challenges for healthcare professionals, particularly nurses. This study examines psychiatric nurses’ perceptions of violence and its impact on the quality of care they provide.

**Objectives:**

The primary objective is to assess the influence of violence on the quality of care in psychiatric settings, with a focus on the experiences and perspectives of nurses.

**Methods:**

We employed a questionnaire-based survey administered to 30 psychiatric nurses working in both inpatient and outpatient psychiatric units of the Razi hospital Manouba. The survey gathered information on the prevalence of violence, types of violence encountered, and the impact on nursing practice.

**Results:**

Of the 30 respondents, 75% identified as female and 25% identified as male. Most of them had more than five years of experience. The primary results revealed that all the psychiatric nurses reported experiencing at least one incident of violence during their psychiatric nursing careers. Regarding exposure to verbal violence, the results indicated that 52% encountered it sometimes, 22% often, 17% very often. Regarding physical violence, 30% experienced it rarely, 26% sometimes, 13% often, and 13% very often. For sexual violence, 56% reported never experiencing it, 8% rarely, 26% sometimes, and 8% very often. These incidents had varying effects on nurses’ emotional well-being, job satisfaction, and the quality of care they were able to provide. 53% of nurses reported experiencing emotional distress and feelings of anxiety as a result of violence, 13% felt anger and frustration. One nurse declared he was not affected emotionally. Most of the respondents (75%) indicated that their job satisfaction had been negatively affected by violent incidents. 40% of respondents stated that violence has a negative impact on their relationship with patients, but they make efforts to maintain care quality. Whereas, 20% found ways to strengthen connections despite challenging experiences.The most commonly endorsed strategies to cope with violence included attempting to master their emotions by remaining calm and patient (78% of respondents), seeking assistance or the presence of other healthcare team members (65%), and maintaining a safe distance from patients (69%). Fewer participants reported raising their voice and adopting a position of authority (30%), while a minority indicated engaging in additional training on the management of violent situations (20%). These results illustrate the diverse range of personal coping strategies.

**Conclusions:**

Violence in psychiatric settings has a multifaceted impact on psychiatric nurses, affecting both their emotional well-being and the quality of care they provide. Strategies for managing and preventing violence, as well as supporting nurses in coping with these challenges, are essential for maintaining high-quality psychiatric care.

**Disclosure of Interest:**

None Declared

